# Three-Dimensional Measurement Method of Four-View Stereo Vision Based on Gaussian Process Regression

**DOI:** 10.3390/s19204486

**Published:** 2019-10-16

**Authors:** Miao Gong, Zhijiang Zhang, Dan Zeng, Tao Peng

**Affiliations:** Key Laboratory of Specialty Fiber Optics and Optical Access Networks, Joint International Research Laboratory of Specialty Fiber Optics and Advanced Communication, Shanghai Institute for Advanced Communication and Data Science, Shanghai University, 99 Shangda Road, Shanghai 200444, China

**Keywords:** multisensor system, Gaussian process regression, Bayesian reasoning method

## Abstract

Multisensor systems can overcome the limitation of measurement range of single-sensor systems, but often require complex calibration and data fusion. In this study, a three-dimensional (3D) measurement method of four-view stereo vision based on Gaussian process (GP) regression is proposed. Two sets of point cloud data of the measured object are obtained by gray-code phase-shifting technique. On the basis of the characteristics of the measured object, specific composite kernel functions are designed to obtain the initial GP model. In view of the difference of noise in each group of point cloud data, the weight idea is introduced to optimize the GP model, which is the data fusion based on Bayesian inference method for point cloud data. The proposed method does not require strict hardware constraints. Simulations for the curve and the high-order surface and experiments of complex 3D objects have been designed to compare the reconstructing accuracy of the proposed method and the traditional methods. The results show that the proposed method is superior to the traditional methods in measurement accuracy and reconstruction effect.

## 1. Introduction

The combination of structural illumination and stereo vision has recently provided increased possibilities for three-dimensional (3D) object measurement, robot vision, and mechanical device control [1,2,3]. Stereo vision measurement methods can be divided into monocular stereo, binocular stereo, and multivision stereo (MVS) methods [4,5,6]. In comparison with the binocular stereo measurement technology, MVS can enlarge the single measurement range, and the multicameras capture the shape of the object during the measurement process at a certain moment instantaneously; thus, MVS is not only suitable for measuring static and nonstrict static objects but also for dynamic and real-time online measurement.

The measurement plan of four-view stereo measurement method in this study is one of the applications of MVS technology. For traditional multisensor working independently in its system, Wu et al. presented a flexible 3D reconstruction method based on phase matching, which reduced the complexity of calibration between single-sensor systems [7]. Xue et al. presented an improved patch-based multiview stereo method by introducing a photometric discrepancy function based on a DAISY descriptor; this method obtains good reconstruction results in occlusion and edge regions of large-scale scenes [8]. Zhang et al. created a multiview stereo vision system for true 3D reconstruction, modeling, and phenotyping of plants. This system yielded satisfactory 3D reconstruction results and demonstrated the capability to study plant development where the same plants were repeatedly imaged and phenotyped over time [9]. The entire process of stereo vision system could be realized in a single field-programmable gate array to improve the real-time performance of the system [10,11,12,13,14].

Given the diversity of measurement objects, multiscale surfaces may have completely different topographical features at different scales, which makes establishing a high-precision model difficult. Gaussian process (GP) is a machine learning algorithm based on Bayesian inference models with strict theoretical foundation basis of statistical learning [15,16]. GP has good adaptability in handling complex problems, such as high-dimensionality and linear inseparability, and has strong generalization capability. In comparison with neural networks and support vector machines, GP has the advantages of easy implementation, and adaptive acquisition of hyperparametric and flexible nonparametric inference [17,18]. Given these advantages, the GP regression (GPR) algorithm has developed rapidly in recent years and has obtained numerous research results; however, most of the research is basically limited to the application of simple geometry, which cannot solve the problems related to multiscale complex surfaces [19,20,21,22,23,24,25]. The traditional GPR algorithm assumes that the entire sampling process is affected by independent Gaussian covariance noise, which makes the derivation of the GPR model simple and feasible [26,27]. However, in the actual measurement process, the noise of different observation points has different variances because of reason, such as the change of sensor environment, and the research of Gaussian heteroscedastic noise has attracted increasing attention [28,29,30].

In view of the advantages and disadvantages of the aforementioned MVS and GPR methods applied in practical measurement, a 3D measurement system of four-view stereo vision based on GPR is proposed in this study. First, two sets of point cloud data with different characteristics are obtained by using the gray-code phase-shifting technique to model the measured object. A special composite kernel function is designed on the basis of the surface morphological characteristics of the measured object to establish the initial GP model. Then, the weighted idea is introduced into the point cloud noise model, and the GP model is optimized by Bayesian reasoning method. This method does not require complicated parameter transfer in camera calibration, which reduces the superposition of systematic errors. The diversity of composite kernel functions makes the application of the method extensive and effective.

## 2. Measurement Principle

### 2.1. Measurement System

**Figure 1:**
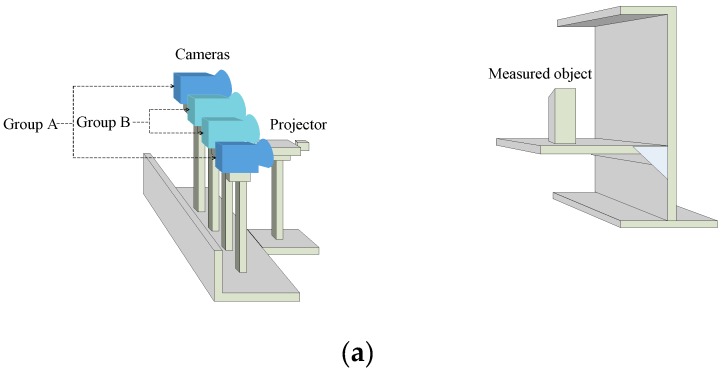

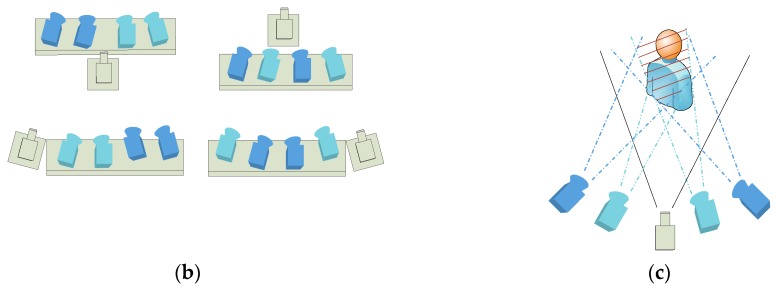
(**a**) System structure; (**b**) sensor layout; and (**c**) common visual field.

The schematic view of the optical setup for the 3D measurement system is shown in Figure 1. This system comprised of a projector and four cameras. These cameras were modeled as a traditional pinhole model, and the projector projects striped, structured light images. The four cameras in this system could be divided into two binocular systems according to camera type. However, the relationships between the cameras, and the cameras and the projector were varied and not necessarily the same as Figure 1a. It can also be the layout in Figure 1b; is not limited to the four types, as long as the “common visual field” (i.e., the shaded portion in Figure 1c) satisfies the measurement requirements.

When measuring the same object, a correlation existed between the measured data from the two groups of cameras. Given the different hardware parameters of the camera, the characteristics of the measurement data were different. The camera with more accurate data was labeled Group A, and the camera with a larger density of data is labeled Group B. Therefore, the 3D reconstruction problem of this study could be formulated into a condition estimation problem, which could fuse the information from the data sets of Groups A and B to improve the quality of reconstruction.

### 2.2. Measurement Principle

The measurement principle of binocular vision is similar to the stereo vision of human eyes. During the measurement process, a series of binary structured light patterns was projected to the object via the projector, and while being captured by the cameras. The two sets of images were encoded and decoded separately to obtain their phase information and were corrected with the constraints of the binocular vision polar; then, dense stereo matching was performed to search for the homologous point. Finally, the 3D point cloud data were obtained using a triangulation method to obtain the disparity between homologous points.

**Figure 2:**
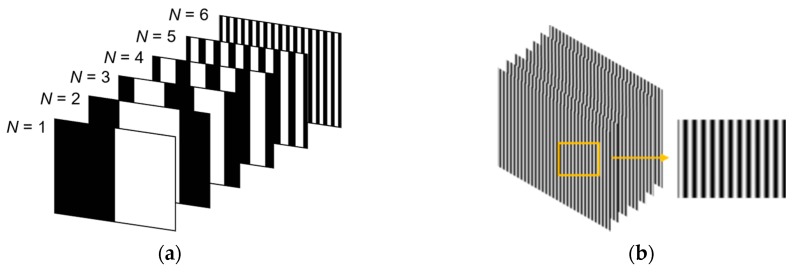
Structured light grating; (**a**) the gray code fringe pattern; and (**b**) the phase-shifting pattern.

The details of the gray-code phase-shifting method are as follows. First, the digital light processing (DLP) projector projects six gray-code black-and-white fringe patterns to the measured object (Figure 2a). In the image captured by the camera, the measured object was marked with a black stripe coverage area as 0 and a white stripe coverage area as 1. The measurement range could be marked from 000000 to 111111, which could be divided into 26 segments. After decoding the gray code, each segment could obtain a periodic number between 0 and 63. Then, a seven-step phase shift method was used, in which the projected phase-shifting pattern was shifted one-seventh period in turn in the vertical direction. The left side of Figure 2b was the seven-step phase-shifted patterns, and the right side was a partial enlargement view of the phase-shifted pattern. The phase-shifted pattern could subdivide the segments of the gray-code segmentation. After decoding the phase-shifted image sequence, the points in the section marked by the gray-code image sequence could obtain the phase principal value in the interval [0,1]. Each point in the measured range could be represented by an absolute phase value by combining the periodic number value with the phase principal value.

## 3. Optimization Method

### 3.1. GP

As mentioned in Section 1, the measurement data of the system were inevitably associated with uncertainty. This uncertainty is generally the result of the combination of sensor noise, calibration, and localization and is fundamental to perform GPR. GPR is a recently developed machine learning regression approach. It is a nonparametric kernel-based probabilistic model and a Bayesian regression model [20,31].

GP is a set of random variables in which the linear combination of any finite number of samples has a joint Gaussian distribution. It can be completely specified by a mean function m(X) and an n × n covariance matrix K(X, X) [15], as shown as follows:

(1) f(X)~N(m(X),K(X,X)).

Generally, before the measurement, prior knowledge about the surface geometry is lacking; thus, m(X) is usually set to a zero-offset mean function. The form of the regression problem can be defined as [32]:

(2) y=f(x)+ε,

where y: R→R is the measured data, and y = [y_1_, y_2_, …, y_n_]. ε~N(0, σ^2^) is the associated measurement error at x and x ∈ X ⊆ R. Assume that (x_i_, y_i_) for I = 1, …, n is the training data. “f()” is the unknown function, and this study aimed to make an inference about this function by using (x_i_, y_i_) observations. 

The prior distribution of y is:

(3) $$y\sim N(0,K(X,X)+\sigma_{\varepsilon }^{2}I).$$

The relationship among the data in GP is modeled by a covariance matrix. Therefore, the prediction of f* at arbitrary position x* can be obtained from the joint distribution of f* with y, as shown as follows:

(4) $$\left[\begin{array}{l} y\\ f^{*} \end{array}\right]\sim N(0,\left[\begin{array}{cc} K(X,X)+\sigma_{\varepsilon }^{2}I & K(X,x^{*})\\ K(x^{*},X) & K(x^{*},x^{*}) \end{array}\right]),$$

where x = [x_1_, x_2_, …, x_n_] is the location of the measured data, I is the identity matrix, and σ_ε_ is a hyperparameter that represents the noise variance associated in y. K(X, X) = K_n_= (k_ij_) is an n × n covariance matrix. K_ij_ = k(x_i_, x_j_) is used to measure the correlation between x_i_ and x_j_. K(X, x*) = K(x*, X)^T^ is an n × 1 covariance matrix between test point x* and training set X. K(x*, x*) is the self-covariance of x*.

Then, the mean m* and the variance cov(f*) of f* can be obtained from the marginal distribution of f*, as shown as follows:

(5) $$f^{*}|x^{*},X,y\sim N(m^{*},\mathrm{cov}(f^{*})),$$

where

(6) $$m^{*}=K(x^{*},X){\left(K(X,X)+\sigma_{\varepsilon }^{2}I\right)}^{-1}y,$$

(7) $$\mathrm{cov}(f^{*})=K(x^{*},x^{*})-K(x^{*},x^{*}){\left(K(X,X)+\sigma_{\varepsilon }^{2}I\right)}^{-1}K(X,x^{*}).$$

### 3.2. Covariance Kernel Function Selection

The covariance kernel function contains the assumptions about the function to be studied and represents the correlation between the observations. Therefore, GP modeling is basically an inference process for determining an appropriate kernel function. The most common basic kernel functions are the square exponential (SE), Matern class (MC), periodic (PER), and rational quadratic (RQ) functions. These basic kernel functions have their own characteristics, which provide a great deal of flexibility in modeling different types of surface topography [15,20].

The SE kernel function obtains the value of covariance by calculating the space distance between two data points, and it has a strong interpolation capability. The formula for SE kernel function is:

(8) $$K_{SE}(x,x^{\prime })=\theta^{2}\exp(-\frac{|x-x^{\prime }{|}^{2}}{2l^{2}}),$$

where, hyperparameter θ controls the scale of the model. (x − x′) and hyperparameter l are characteristic length dimensions, and l is the form of the model.

In comparison with the SE kernel function, the RQ kernel function is more conducive in catching the sudden change in the function, and its form can be expressed as follows:

(9) $$K_{RQ}(x,x^{\prime })=\theta^{2}{\left(1+\frac{{\left(x-x^{\prime }\right)}^{T}(x-x^{\prime })}{2\alpha l^{2}}\right)}^{-\alpha },$$

where α > 0. RQ kernel can be regarded as the sum of SE kernels with different scaling lengths. When the value of a is larger, the sampled function tends to be sampled by the SE kernel function.

The MC kernel function can be used to fit the short-term irregular time series data well. The formula is as follows:

(10) $$K_{MC}(x,x^{\prime })=\frac{2^{1-v}}{\Gamma (v)}\exp{\left(\frac{\sqrt{2v}|x-x^{\prime }|}{l}\right)}^{v}K_{v}(\frac{\sqrt{2v}|x-x^{\prime }|}{l}),$$

where, p is a positive integer; “Γ()” is the standard Gamma function; “*Kv()*” is the modified second-order Bessel function; and v = (p + 1)/2, which is an additional hyperparameter that controls the degree of the differentiability of the surface models, such that they are only v + 0.5 differentiable. Hence, this kernel function is suitable for modeling rough surface topography.

If the periodic priori hypothesis must be added to the regression model, then the following PER kernels can be applied:

(11) $$K_{PER}(x,x^{\prime })=\sigma_{f}^{2}\exp(-\frac{2}{l^{2}}\sin^{2}(\pi \frac{\left\|x-x^{\prime }\right\|}{p})),$$

where the period length of the function to be approximated is expressed by the hyperparameter p. When p takes different values, the sampled functions have different periods.

SE, MC, and RQ are all local kernel functions that have strong nonlinear approximation capability; however, their generalization capability is weak. The PER kernel function is a global kernel function that has a strong generalization capability; however, its data learning capability is worse than the local kernel function. Each kernel function can only solve one type of problem and cannot be used to satisfy all problems. Therefore, on the basis of the prior knowledge of the actual surface topography of the measured object, several kernels with different attributes can be selected and combined accordingly, that is, using complex kernels for geometric modeling. Generally, the additive kernel function has good prediction performance. The multiplicative kernel function has improved flexibility, which makes it uncertain outside the sampling points, and it cannot accurately predict the shape of the original measured object.

**Figure 3:**
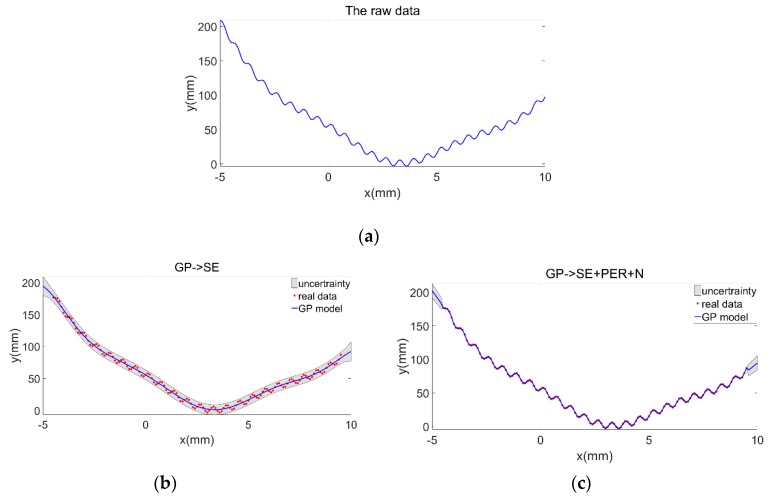
Gaussian process (GP) modeling of a multifeatured curve. (**a**) Raw data model of the curve; curve model after GP regression (GPR) with (**b**) square exponential (SE), and (**c**) SE + periodic (PER) + Noise.

Figure 3 takes a multifeature geometric curve with a small-scale shape of sinusoidal waveform superimposed on a large-scale curve g as an example. The formula of curve g is g = (5x/π − 6)^2^ + 10(1 − 1/8π)cosx + 10, which has 150 × 150 sampling points with an interval from −5 mm to 10 mm. Figure 3a shows the raw data model of this curve. The effect of noise was not considered, Figure 3b,c show the GP model of this curve established by single kernel function SE and composite kernel function (SE + PER + Noise), respectively. The shape and the estimation uncertainty of the reconstructed model show that GP with SE kernel function only successfully modeled the general trend of the curve, whereas GP with (SE + PER + Noise) kernel function not only successfully modeled the general trend of the curve but also successfully modeled the details of the small term variation. The result indicates that the GP model could estimate the uncertainty of the model outside the observation area. When the estimation is far away from the observation area, the covariance becomes weaker and the associated uncertainty becomes larger.

### 3.3. GP-Based Sampling and Data Fusion

Two sets of measurement data were available in this study. The data set of Group A was low-density and high-quality, which was the opposite of that of Group B. The data sets of the two measurement systems in Section 2.2 must be properly processed for data fusion to achieve a suitable 3D reconstruction effect. Data fusion is not a simple data overlay; that is, it can be divided into two key steps, namely, registration and fusion. Generally, except for repeated measurements, all fusion methods for spatial data must be registered prior to fusion, such that different data sets can be linearly transformed into the same coordinate system [24]. In this study, the data sets are initially roughly matched and then further registered and refined by the iterative closest point procedure (ICP), and fusion should be conducted on the data points within the overlapping areas of the registered data sets. 

A large amount of point clouds will cause a heavy burden on computation; thus, data sets should be preprocessed before data fusion, that is, point cloud simplification and sampling [33]. Typically, the sample must cover the entire model as much as possible, because no matter the specific algorithm used to process the model data set, the measurement uncertainty of the uncovered area of the sample will still be large. However, given that GP can predict outside the observation area and provide credibility for predictions, few sampling points can be used for data fusion, which improves the efficiency and measurement accuracy of the algorithm. The two data sets obtained the same object’s data; hence, they had the same trend, and only the local details were different. Therefore, they could be represented by the same kernel function and hyperparameters, and only different noise parameters were used to simulate their random errors.

**Figure 4:**
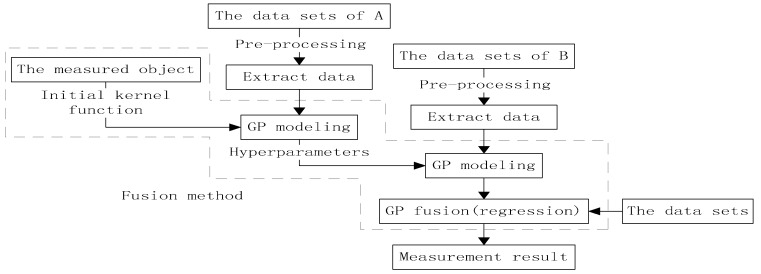
Framework of data fusion.

As show in Figure 4, on the basis of the surface morphological characteristics of the measured object, GP could be used to select specially designed composite kernel functions to model the discrete points of Group A’s data sets. Moreover, optimized hyperparameters were obtained, which were used as the prior input of GP modeling for Group B’s data sets, and only the noise parameters were optimized. The GP models obtained by the two models had the characteristics of the two data sets. Finally, the data of the target area were fused and predicted to obtain the final 3D measurement model. 

This fusion method can be expressed as [34]:

(12) $$\left[\begin{array}{l} y^{\prime }\\ f^{\prime *} \end{array}\right]\sim N(0,\left[\begin{array}{cc} K(X^{\prime },X^{\prime })+diag(\sigma_{\in A}^{2}I,\sigma_{\in B}^{2}I) & K(X^{\prime },x^{\prime *})\\ K(X^{\prime },x^{\prime *}) & K(x^{\prime *},x^{\prime *}) \end{array}\right]),$$

where y′ = [y_A_, y_B_] are the data sets of Groups A and Group B; X′ = [X_A_, X_B_] are the location of data sets; and σ_A_^2^ and σ_B_^2^ the noise parameters of Groups A and B, respectively. In view of Section 2.1, the mean and uncertainty of the model after fusion can be determined as:

(13) $$m^{*}(X^{*})=m^{*}(X_{A})+K(X^{*},X^{\prime })\omega [y_{A},y_{B}],$$

(14) $$\mathrm{cov}(f^{*}(X^{*}))=\mathrm{cov}(f^{*}(X_{A}))-K(X^{*},X^{\prime })\omega K(X^{\prime },X^{*}),$$

(15) $$\omega =\left[\begin{array}{cc} K_{AA}^{-1}K_{AB}KK^{-1}K_{BA}K_{AA}^{-1} & -K_{AA}^{-1}K_{AB}\tilde{K^{-1}}\\ -KK^{-1}K_{BA}K_{AA}^{-1} & \tilde{K^{-1}} \end{array}\right],$$

(16) $$KK=K_{BB}-K_{BA}K_{AA}^{-1}K_{AB},$$

(17) $$\left\{ \begin{array}{l} K_{ii}=K(X_{i},X_{i})+\sigma_{i}^{2}I\\ K_{ij}=K(X_{i},X_{j}) \end{array}\right.i,j\in \{A,B\},$$

where σ_i_ represents the noise of the *i*th data set.

## 4. Experimental and Discussion

### 4.1. Simulation Examples and Analysis

The curve in Section 2.2 was taken as an example to verify the effectiveness of the proposed method (Figure 3a), and the higher-order surface z = $\sqrt{10}$ (x^2^/25 + y^2^/25 − 3) + (y^2^/25 − 3)(x^2^/100 − y^2^/100), which has 150 × 150 sampling points ranging from −10 mm to 10 mm was taken an example for the analysis.

**Figure 5:**
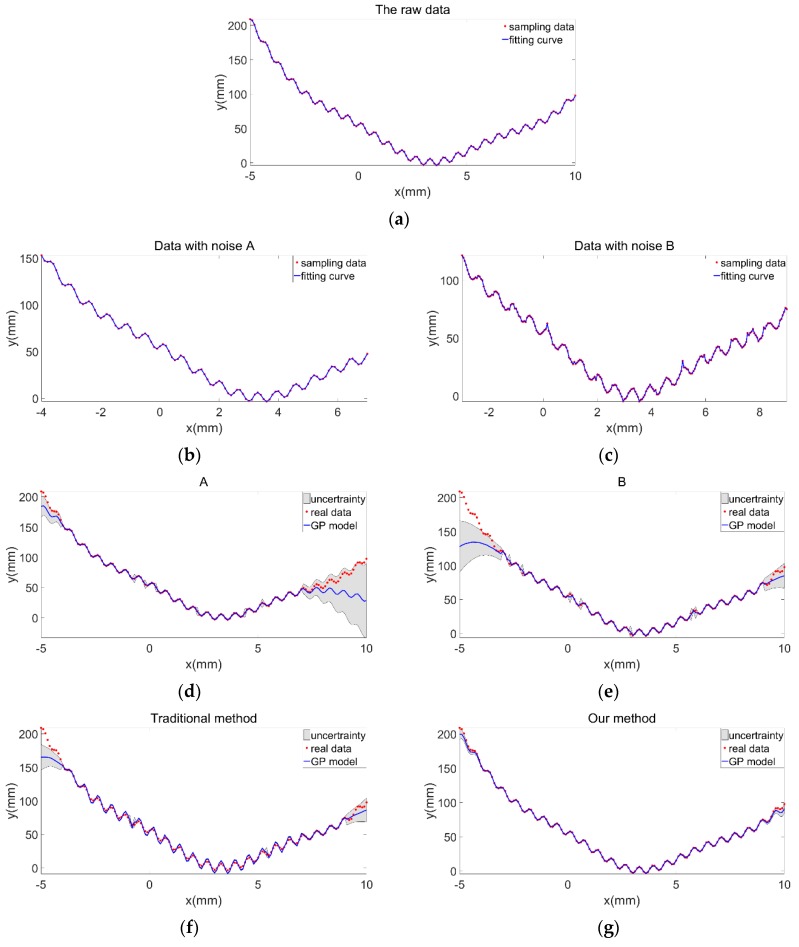
(**a**) Raw data of the curve; sampling data with (**b**) noise A, (**c**) noise B; GPR modeling of curves using (**d**) sampled data with noise A, (**e**) sampled data with noise B, (**f**) the traditional method, and (**g**) the proposed method.

In the reconstruction of the curve, sampling was performed twice. The two groups of sampled data had different measurement ranges and densities. The first sampling range was [−4, 7] with an interval of 0.1, and the second sampling range was [−3, 9] with an interval of 0.05. Then, Gaussian white noise A: ε_A_~N(0,σ_A_^2^), σ_A_^2^ ∈ [0.5, 1] and B: ε_B_~N(0,σ_B_^2^), σ_B_^2^ ∈ [1.5, 3] were added to the two sets of sampled data. The sampling results are shown in Figure 5b,c. The fitting curves of the two sets of sampling data had the same trend; however, the details of the curves were slightly different due to the influence of noise.

Figure 5e show the curve models modeled using only one set of sampled data. This method had low precision and weak prediction capability for unsampled regions. Figure 5f shows the curve model obtained using a traditional method. The two sets of sampled data were used together as training samples. The amount of information became large. However, the idea of weight was not introduced into the algorithm, such that the influence of noise in the samples still existed. Thus, the modeling accuracy was low, and the unsampled area could not be predicted well. Figure 5g shows the curve model obtained by the proposed method. The method assigns corresponding weights to various noises; thus, the noise was effectively suppressed. At the same time, the effective data of the two sets of sampling models were well extracted, which improved the prediction accuracy of the unused areas.

**Figure 6:**
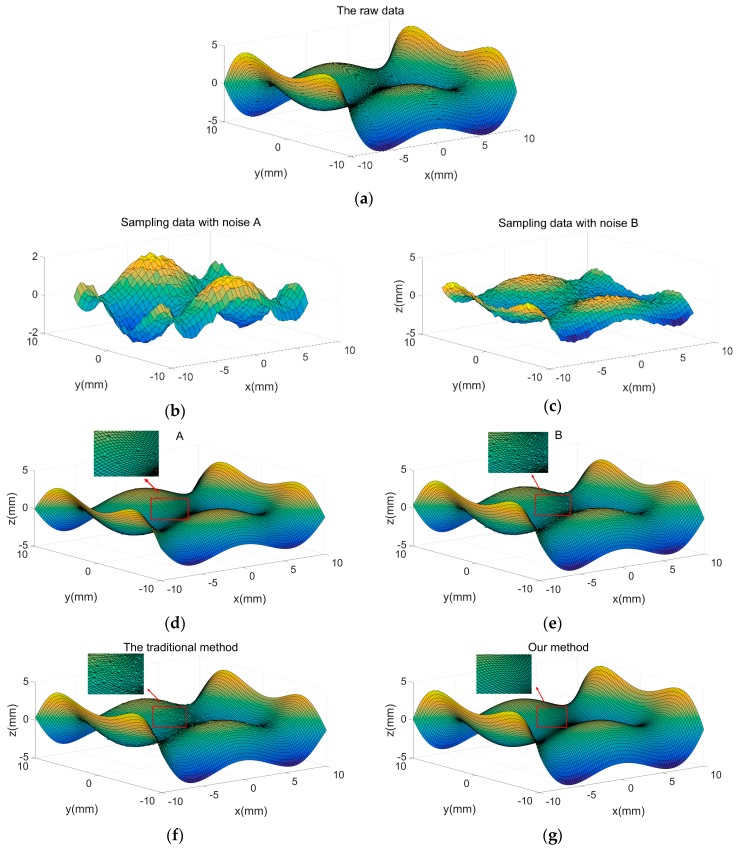

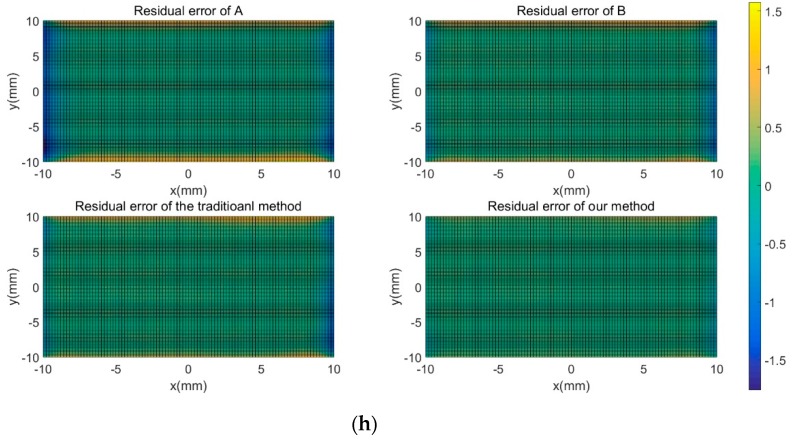
Higher-order surface. (**a**) Raw data; sampling data with (**b**) noise A, and (**c**) noise B; GPR modeling using (**d**) sampled data with noise A, (**e**) sampled data with noise B, (**f**) the traditional method, and (**g**) the proposed method; and (**h**) errors in reconstruction using the four methods.

In the reconstruction of the higher-order surface, similar to the curve reconstruction, two samples with different measurement ranges and densities were taken. The first sampling range was [−8, 8] with an interval of 0.5, and the second sampling range was [−9, 8.5] with an interval of 0.3. Then, Gaussian white noise A: ε_A_~N(0,σ_A_^2^), σ_A_^2^ ∈ [0.01, 0.08] and B: ε_B_~N(0,σ_B_^2^), σ_B_^2^ ∈ [0.06, 0.1] were added to the two sets of sampled data. The results are shown in Figure 6b,c.

Figure 6d,e show the reconstruction models obtained by regression only using a single set of sampled data. The reconstructed surface had varying degrees of noise, and the sampled part in the edge region of the model had large errors using the original data model. Figure 6f shows the reconstruction model obtained by regression using traditional methods. The surface of the model was still noisy, and the predictability of the edge was not sufficiently good. Figure 6g presents the 3D model obtained using the proposed method. The results show that this method could effectively suppress noise and had a high prediction accuracy for the unsampled edges of the model. Figure 6h shows the residual error graph of the reconstructed model and the raw model obtained by the four methods. Evidently, the proposed method had advantages. 

### 4.2. Experimental Preparation

**Figure 7:**
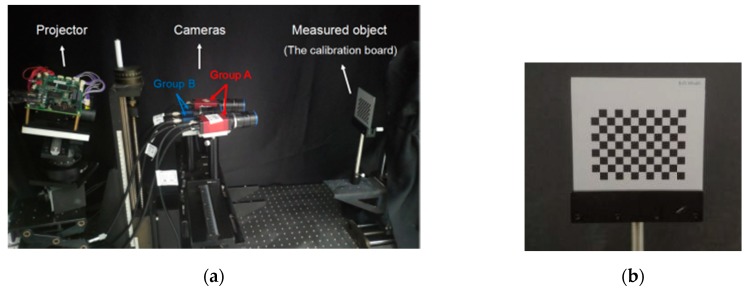
(**a**) Experimental setup. (**b**) Calibration board.

An experimental platform was set up in the laboratory to verify the feasibility of the proposed measurement method. This multicamera measurement system consisted of four cameras and one projector. The experimental setup is shown in Figure 7a. The camera model of Group A was Manta G-504 with resolution of 2452 × 2056 and pixel size of 3.45 μm. The camera model of Group B was MER-500-14GM/C-P with resolution of 2592 × 1944 and pixel size of 2.2 μm. The model of the camera lens was OPT-165M. The projector was DLP Light Crafter 4500. Figure 7b shows the calibration board with a size of 100 mm × 100 mm.

### 4.3. Experimental Results and Analysis

**Figure 8:**
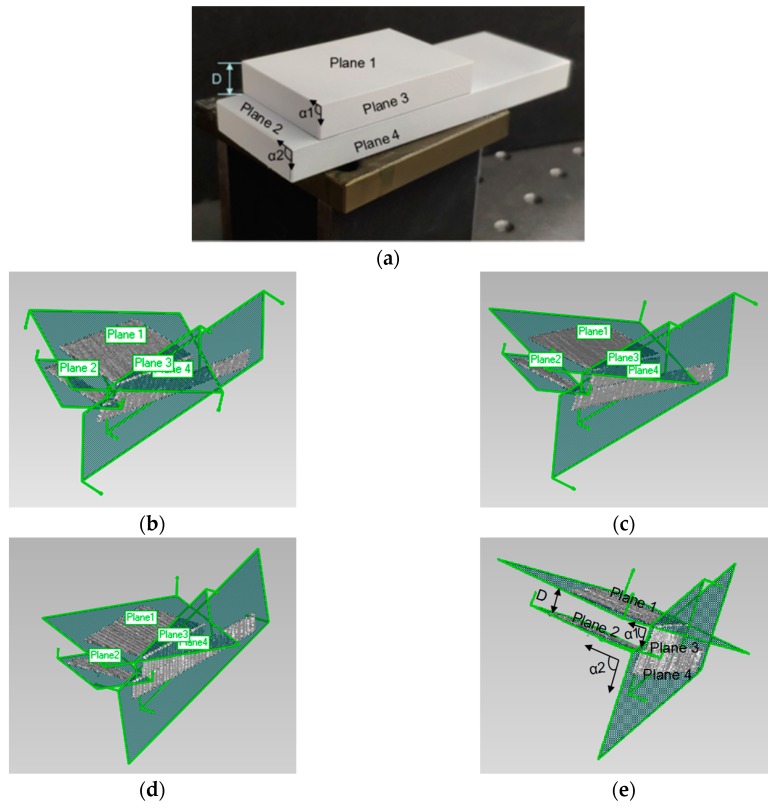
Standard object measurement experiments: (**a**) standard measurement object; point cloud and fitting planes of (**b**) Group A without GPR, (**c**) Group B without GPR, (**d**) the traditional method, and (**e**) the proposed method.

A standard object with two steps was used as the measurement object in Figure 8a to verify the accuracy of the system and obtain the measurement errors. The object was randomly placed in the measurement area. The distance between steps was accurately measured (averaged by multipoint measurement using digital micrometer), as well as the angles of the apex angle on one side of these two steps (averaged by multiple measurements using digital protractor). Through the obtained internal and external parameters of the cameras, the point clouds of planes 1–4 measured by Groups A and B could be obtained separately, and these point clouds could be fitted by Geomagic, as shown in Figure 8b,c. Figure 8d,e show the fitting results of the point cloud obtained by the traditional method and the proposed method, respectively. Each measurement index was marked on Figure 8e.

The fitted planes were set as the truth, and the distance between planes 1 and 2 and the angle between planes 1 and 3 and between planes 2 and 4 could be calculated according to the obtained plane equation. The measured results obtained by Groups A and B and a two fusion model were compared with the actual values, as shown in Table 1 (the results are the average of multiple measurements). The result shows that our system had good stability and the accuracy of the 3D model fused by the proposed method was improved.

**Figure 9:**
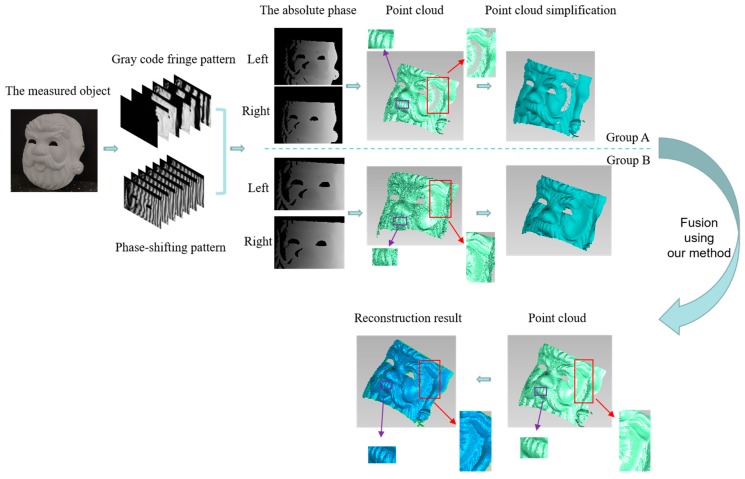
Algorithm flow chart with the Santa Clause mask as an example.

After the measurement accuracy was determined, further experiments were needed to prove the reliability of the proposed method by measuring multiple objects with complex shapes. Some objects, including a Santa Claus mask and Marseille Statue, were measured. To make the experimental process clear, the Santa Claus mask was taken as an example. The entire experiment was shown in Figure 9. The projector was used to project grating patterns onto the object, and the four cameras collected images simultaneously. Subsequently, the phase information of the object could be calculated on the basis of the phase-shifting method to obtain the unwrapped phase. Finally, the 3D coordinates of the object were obtained by phase matching, and their 3D models were displayed in Geomagic. Group A had 1,290,181 points in the original point clouds and 29,280 points after point cloud simplification, whereas Group B had 1,967,616 points in the original point clouds and 32,033 points after simplification. On the basis of the characteristics of the model and the knowledge introduced in Section 3, the composite kernel function SE + MC + Noise, which was composed of the sum of SE and MC functions, was selected to fuse the two models.

The results show that the measurement range of Group A was larger than that of Group B, and the purple rectangular frame indicates that the data accuracy of Group A was higher. In the red rectangular frame, few point clouds were found on the left face of the mask of Group A, and holes appeared when the polygon surface was generated. However, more point clouds were observed at the corresponding positions of Group B. The fused point cloud model contained 2,057,192 points. The fused model combined the advantages of Groups A and Group B. The surface texture was clear, and the point cloud density was high. The polygon patch model of the reconstruction model could intuitively indicate the effectiveness of the proposed method.

**Figure 10:**
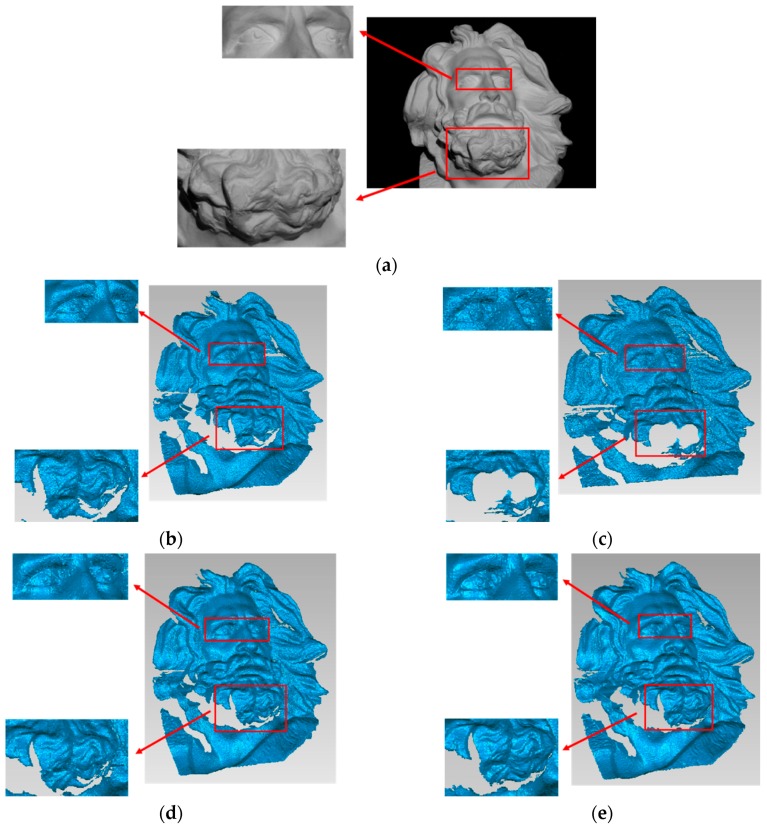
(**a**) Plaster statue of Marseille; reconstruction results of (**b**) Group A without GPR, (**c**) Group B without GPR, (**d**) the traditional method, and (**e**) the proposed method.

The 3D results of the Marseille Statue are shown in Figure 10, and the corresponding enlarged details are provided. The reconstruction result obtained by the proposed method was more consistent with the actual measured object. For example, in the eyes part, the result of the proposed method combined the detailed information of Group A and the contour information of Group B, which results in a clearer and more accurate outcome. In the beard part, the results of the proposed method fully considered the complementarity of point cloud information of the two models and used the predictability of GPR to restore the unknown region with high accuracy, which made this part richer and fuller.

The above experiments further verified the reliability and good characterization performance of the proposed method. This method fully utilized the distribution and correlation information of the data obtained by the two measurement systems. It was also capable of robust measurement and reconstruction of the 3D shape of complex surface objects. In comparison with the traditional methods, the proposed method was more stable and had better reconstruction accuracy.

## 5. Conclusions

A GPR fusion method that combines high-density, low-quality data with low-density, and high-quality data was presented in this study. This method could measure free-form objects and without complex camera-projection calibration and parameter transfer. The main characteristics of the proposed approach can be summarized as follows. (i) The relative positions among the cameras and between the cameras and the projector have no strict requirement, which makes measurement easy. (ii) The fusion fully considers the correlation between two data sets, which makes the 3D information of the fused model rich and accurate. (iii) The predictability of the algorithm for unmeasured 3D information makes the final reconstruction model complete.

Simulations on the curve and the high-order surface were developed to compare the reconstructing accuracy of the proposed method and traditional methods. The results show that the reconstruction accuracy of the proposed method was far superior to the traditional methods. Experiments on complex 3D objects were also carried out in this paper, which further confirmed the superiority and stability of this method in measuring accuracy and reconstruction effect compared with traditional methods.

The quality of the final 3D model was closely related to the preprocessing of the data sets before fusion. In a future study, we will focus on the simplification and sampling of scattered point clouds to improve the accuracy and efficiency of the method.

## Figures and Tables

**Table 1:** Summary of measurement results (the averaged of multiple measurements).

	Actual Value	Measured Value			
		Group A	Group B	Traditional Method	Our Method
**D (mm)**	9.023	9.125	9.147	9.094	9.058
Absolute error1 (mm)		0.102	0.124	0.071	0.035
α1 (°)	90.81	90.932	91.017	90.912	90.908
Absolute error2 (°)		0.122	0.207	0.102	0.098
α2 (°)	90.82	90.914	90.993	90.933	90.902
Absolute error3 (°)		0.094	0.173	0.113	0.082
